# Vitamin D deficiency or insufficiency is associated with lower urinary tract symptoms

**DOI:** 10.1590/S1677-5538.IBJU.2021.0645.1

**Published:** 2022-03-01

**Authors:** Sandra Barbosa da Silva

**Affiliations:** 1 Universidade do Estado do Rio de Janeiro Laboratório de Morfometria, Metabolismo e Doenças Cardiovasculares Rio de Janeiro RJ Brasil Laboratório de Morfometria, Metabolismo e Doenças Cardiovasculares. Universidade do Estado do Rio de Janeiro - UERJ, Rio de Janeiro, RJ, Brasil

## COMMENT

The present work presented a very relevant and original assessment associating vitamin D deficiency in children with overactive bladder related urinary incontinence (1). This topic has been studied in adults, but still little explored in children, which makes this study very relevant and current. Overactive bladder (OAB) is the most important underlying cause of urgency that leads to incontinence in men and women (2). In ddition OAB is a syndrome that is associated with multiple urinary tract symptoms and could affect the patient's quality of life. The vitamin D deficiency and insufficiency is reported to be linked to OAB syndrome, which exacerbated by stress conditions. Urinary incontinence and hypovitaminosis D are prevalent problems of the geriatric population (3). However, little is known about its role in pediatric OAB; in this context the results of the present study make an important contribution to guide future evaluation of vitamin D deficiency and considering it in treatment-resistant cases.

Regarding to Vitamin D, his synthesis depends on adequate sunlight exposure (including in tropical countries like Brazil) and also is available from the diet, however, it is biologically inactive. It becomes activated after it undergoes two steps of hydroxylation, in the liver and in the kidneys; Vitamin D is a group of fat-soluble secosteroids responsible for increasing intestinal absorption of calcium, magnesium, and phosphate, and has many other biological effects (4). Vitamin D status is identified through measurement of sérum vitamin D.

Vitamin D deficiency is considered as serum vitaamin D levels of <20 ng/ml, insufficiency is considered as levels ranging between 20 and 30 ng/m and normal levels of vitamin D are considered to be >30 ng/ml. Skeletal and smooth muscle growth and function are reported to be affected by vitamin D status (5). In this context, Vitamin D receptors are found in skeletal and smooth muscle cells throughout the body, specifically in the bladder detrusor muscle (6); Vitamin D is the single most deficient vitamin and musculoskeletal pain and weakness are symptoms found to be accompanied by vitamin D deficiency. The possible effect of vitamin D on the detrusor muscle and on the levator ani muscles, which contain both smooth and striated muscle fibers, could explain the association between vitamin D deficiency and urinary incontinence (6). Previous study have demonstrated that a vitamin D receptor agonist can regulate calcium entry through L-type Ca2+ channels in the human bladder smooth muscle cells. This suggests a possible effect of the vitamin D receptor agonist on the modulation of bladder contractile mechanisms (6).

The present study in its discussion reports that there is an increasing evidence suggesting that vitamin D deficiency or insufficiency is associated with lower urinary tract symptoms (7). In addition, these symptoms may be attenuated after Vitamin D deficiency in children can cause OAB through detrusor muscle activity and impair quality of life, especially by causing urinary incontinence. The Vitamin D intake can reduce symptoms and improve quality of life, as in adults. As far as we know, there is no study in the literature evaluating the relationship between vitamin D and OAB in the pediatric age group.

In the current study, the authors aimed to analyze serum vitamin D levels in children with OAB-related urinary incontinence, to determine their effect on symptoms and quality of life, and to evaluate whether vitamin D supplementation alleviated these symptoms and improved quality of life.

In conclusion, the present study showed that vitamin D deficiency was more common in children with OAB-related urinary incontinence than in healthy children, and demonstrated that vitamin D supplementation could reduce urinary symptoms associated with OAB and improve quality of life in treatment-resistant cases. The findings reflect the importance of evaluating vitamin D levels in children with OAB-related urinary incontinence. Although vitamin D deficiency is not routinely evaluated in every patient, they suggest considering it in treatment-resistant cases.

These findings are relevant and encouraging as it is not difficult to assess serum vitamin D levels, as supplementation is easy to perform in children, the previous studies cited throughout this commentary and the unpublished results of the present study in children have demonstrated and encouraged that vitamin D supplementation improves quality of life with the improvement of symptoms and that vitamin D supplementation will increase the treatment response in treatment-resistant cases by supporting other treatment modalities.
